# Obituary

**Published:** 1920-03

**Authors:** 


### OBITUARY

Henry W. Morgan, M.D., D.D.S. Born October 25, 1853; died January 17, 1920. Educated in Nashville H. S. Graduated from Vanderbilt Medical School, February 25, 1875; Philadelphia Dental College, February 25, 1876. Began practice of dentistry with his father, W. H. Morgan, 1876. Began teaching in Vanderbilt University Dental Department, 1879, as assistant to professor of operative dentistry. Married to Matilda Alloway Evans, 1880.

Treasurer National Association of Dental Faculties and American Dental Association. President National School of Dental Technics, National Association of Dental Faculties, 1902. Dean Dental Department Vanderbilt University, 1911. Supreme Grand Master Delta Sigma Delta Supreme Chapter, 1918. Trustee Walden University, 1914. President Board of Trustees Maharry Medical and Dental College, 1916. Member Royal Arcanum, Knights of Pythias, thirty-third degree Mason, etc. Member of numerous literary and scientific societies and of all the national dental societies and his own state society. A frequent contributor to dental literature.

---

B. Holly Smith was born in Prince George County, Maryland, March 17, 1858. His father was a clergyman and gave his personal attention to his son's education. He attended the Baltimore College of Dental Surgery and graduated in 1881. Two years later he graduated from the College of Physicians and Surgeons of Baltimore. He taught operative dentistry and surgery in Baltimore College of D. S. and also gave lectures in dental materia medica and dental surgery in the Medical College of P. S. He has been given honorary membership in many local and state societies and has held the important office in all of the national societies, the San Francisco Dental Congress, the Jamestown Dental Convention, the National Faculties Association, Delta Sigma Delta and numerous societies and social clubs in his native city. He has been a frequent contributor to dental literature and discussed essays contributed to dental societies generally.

